# The Effect of Red & Blue Rich LEDs vs Fluorescent Light on Lollo Rosso Lettuce Morphology and Physiology

**DOI:** 10.3389/fpls.2021.603411

**Published:** 2021-02-18

**Authors:** Laura Cammarisano, Iain S. Donnison, Paul R. H. Robson

**Affiliations:** ^1^IBERS, Aberystwyth University, Aberystwyth, United Kingdom; ^2^Next-Generation Horticultural Systems, Leibniz-Institute of Vegetable and Ornamental Crops (IGZ), Grossbeeren, Germany

**Keywords:** LED – light emitting diode, fluorescent light, red lettuce, light spectral composition, irradiance, leaf optical properties, leaf structural and functional traits, light adaptation

## Abstract

The challenges of feeding an increasing population, an increasingly urban population and within an increasingly challenging global environment have focused ideas on new ways to grow food. Growing food in a controlled environment (CE) is not new but new technologies such as broad-spectrum LEDs and robotics are generating new opportunities. Growth recipes can be tailored to plant species in a CE and plasticity in plant responses to the environment may be utilized to make growth systems more efficient for improved yield and crop quality. Light use efficiency within CE must consider energy requirements, yield and impacts on quality. We hypothesized that understanding how plants change their morphology and physiology in response to light will allow us to identify routes to make light more efficient for delivery of high-quality produce. We focused on responses to light in Lollo rosso lettuce which produces compact, crinkly and highly pigmented leaves. We compared the spectra of the commonly used artificial light sources in indoor farming (compact fluorescence tubes, FL, and broad-spectrum light-emitting diodes, LEDs) at two irradiance levels (270 and 570 μmol m^–2^ s^–1^). We discovered LEDs (λ_P_: 451, 634, and 665 nm) produced the same amount of produce for half the incident energy of FL (T5). At higher irradiances LEDs produced 9% thicker leaves, 13% larger rosettes and 15% greater carotenoid content. Leaves differed in light absorptance with plants grown under lower FL absorbing 30% less of mid-range wavelengths. We show that the relative efficiencies of LED and FL is a function of the irradiances compared and demonstrate the importance of understanding the asymptotes of yield and quality traits. Increasing our understanding of structural and biochemical changes that occur under different combination of wavelengths may allow us to better optimize light delivery, select for different ranges of plasticity in crop plants and further optimize light recipes.

## Introduction

Incident light provides both energy and information by powering and regulating plant growth and development. The use of focused light treatments, with direct effects on physiological processes, allows fine manipulation of the plant phenotype (Carvalho and Folta, 2015). When absorption of photosynthetically active photons exceeds the photosystems capacity to utilize excitation energy, dissipation of the excess energy is necessary to avoid or to reduce the risk of photooxidative damage. The excess excitation energy can be re-emitted as radiation energy and particularly chlorophyll a fluorescence or as non-radiative energy that can be dissipated thermally via non-photochemical quenching (NPQ) (Niyogi, 2000; Ruban et al., 2007; Kalaji et al., 2017; Kress and Jahns, 2017).

Plants optimize light capture and prevent photodamage in fluctuating light conditions. Adaptations act across different scales from macro scale to the micro scale (Bjorkman and Demmig-Adams, 1995). Adjustments at plant/leaf level affect light interception and absorption through changes in plant compactness, stem elongation, leaf movement, protective pigment synthesis (e.g., anthocyanins), protective leaf layers (e.g., wax or trichomes) and leaf area. Further adjustments include changes in leaf ultrastructure, i.e., the number of cells or airspaces, chloroplast movement and more in-depth changes in photosystem stoichiometry and synthesis of antioxidants to scavenge reactive oxygen species (Bensink, 1971; Štroch et al., 2004; Terashima et al., 2009; Davis et al., 2011).

Not all incident photons are absorbed because of differences in intrinsic absorption levels of different wavelengths of light. This means that, regardless of the amount of light reaching the leaf, the capture of photons and the energy conversion efficacy of radiant energy into biomass depends on the wavelength of the photon (Hoover, 1937; McCree, 1981). Photon energy is inversely proportional to the wavelength (*E* = h c/λ), consequently energy decreases across the electromagnetic spectrum, photons with longer wavelengths (>750 nm) have too little energy for photochemistry (1.8 eV, equivalent to the energy of a red photon) and the short wavelengths photons have excessive energy (Zhu et al., 2008; Barber, 2009; Thapper et al., 2009; Kusuma et al., 2020). Plant adaptive mechanisms to incident radiation can be indicative of light stress, too little or too much, but also include desirable plant quality traits. For instance, in red lettuce, leaf pigmentation is a plant stress response and is an important characteristic for visual and nutritional quality of lettuce (Becker et al., 2014).

Controlled Environment Agriculture (CEA) may produce optimal growth conditions to obtain the best yield all year-round (Kozai, 2013). Plant factories could include environmental stresses in growth recipes to enhance crop quality. Furthermore, plant biochemical and biophysical responses to the environment may change the way light is absorbed and could be exploited to further enhance plant performance (Ustin and Jacquemoud, 2020). Characterizing plant responses, especially at leaf level, to light intensity and spectral quality has great potential for the rapidly evolving indoor farming including environmental optimization of stress application and manipulation of plant morphology (Carter and Knapp, 2001; Carvalho and Folta, 2015; Bergstrand et al., 2016).

The aim of this work was to characterize some of the adaptive morphological and physiological responses to light in the pigmented Lollo rosso lettuce. Morphological responses at plant level (e.g., rosette compactness) and leaf level (e.g., pigmentation, thickness, leaf structural anatomy) were studied in combination with chlorophyll fluorescence and leaf absorptance to investigate plant adaptations to irradiance and light quality. We compared Lollo rosso growing in the same controlled environment (CE) cabinet under two efficient light sources, fluorescent and LEDs, at two irradiances to better understand the relative adaptations and efficacy of the light sources and the interactions between their different light spectra and yield and crop quality.

## Materials and Methods

### Plant Material and Growth Conditions

**FIGURE 1:**
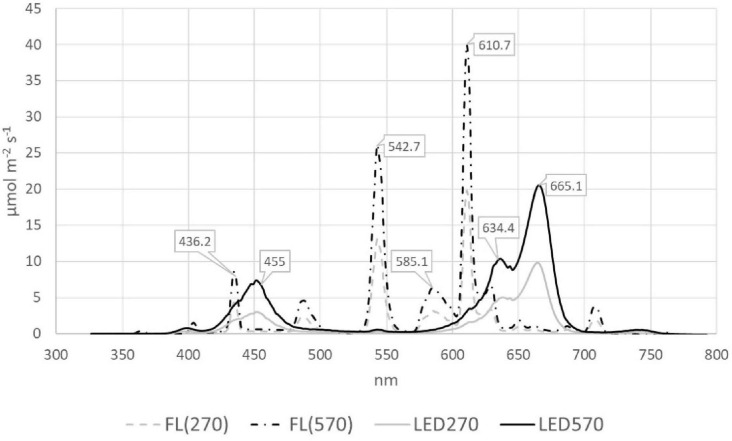
Spectral distribution including peak wavelengths of the four light treatments, FL(270) in dashed gray, FL(570) in dashed black, LED(270) in gray and LED(570) in black. Fluorescent (FL) light provided by T5 fluorescent lamps. LEDs radiation provided by PAR customized LED array (diodes emitting in the blue: 410, 430, and 460 nm and, diodes emitting in the red: 610, 630, and 660 nm).

Red lettuce Lollo rosso seeds (Antonet RZ seeds from RijkZwaan, De Lier, The Netherlands) were sown in 155 g of sieved John Innes No. 3 soil-based compost. Water holding “field” capacity of the compost was calculated following the gravimetric method for soil moisture determination (Reynolds, 1970). Pots (7 cm × 7 cm × 10 cm) were filled, saturated with water, covered with plastic film and left to drain at room temperature (20 ± 5°C). After 24 h pot weight was noted and pots were dried in the oven at 105°C. Every 24 h pots were weighed until stable dry weight (DW) was reached. The dry and wet weights were used to estimate the weight of pots and soil at approximately 0 and 100% field capacity and water content in between these extremes was estimated as a linear proportion of the difference between these values. Pots with plants were individually irrigated to 80% field capacity (205 g) every 48 h until harvest at day 30. The 48 pots containing seeds were placed into the experimental system (Fitotron, growth cabinet) which was partitioned in two halves separated by white reflective sheets (ORCA grow film, California Grow Films LLC). One side of the cabinet was equipped with fluorescent tubes [FL, compact fluorescent tubes spectrum (T5, F28W/835, 3,500 K), with a spectral composition of blue (401–498 nm): green–yellow (499–609 nm): red (610–699 nm): far-red (700–750 nm) of 15: 44: 35: 6%, respectively) and the other half with a photosynthetically active radiation (PAR) customized LED array (EP006, 380–760 nm, Shenzhen Herifi Co., Ltd., China, with a spectral composition of blue (401–498 nm): green–yellow (499–609 nm): red (610–99 nm): far-red (700–750 nm) of 19: 5: 65: 11%, respectively). Two shelves (each shelf was 0.27 m^2^, with 12 plant replicates) were arranged in each side of the cabinet at different heights to generate two irradiance levels (270 and 570 μmol m^–2^ s^–1^) for a total of four light treatments [FL(270) (270 μmol m^–2^ s^–1^), FL(570) (570 μmol m^–2^ s^–1^), LED(270) (270 μmol m^–2^ s^–1^), LED(570) (570 μmol m^–2^ s^–1^)]. Environmental conditions were monitored by four Tinytag Ultra 2 (Gemini Data Loggers, Chichester, United Kingdom) placed in each of the treatment areas. Photoperiod was 18 h, temperature was maintained at an average of 22°C, relative humidity 50% and ambient CO_2_ (environmental data from the individual treatment areas is in Table 1). Irradiance and light spectral composition of the treatments were measured (Figure 1) using the spectroradiometer SpectraPen LM 500 (cosine-corrected, 380–780 nm; Photon Systems International, Drasov, Czech Republic).

**TABLE 1:** Environmental data for the treatments reported.

**Treatments**	**Temperature (°C)**	**Relative humidity (%)**
FL(270)	21.6 ± 0.2	50.1 ± 0.6
FL(570)	22.7 ± 0.1	46.1 ± 0.4
LED(270)	22.0 ± 0.2	49.1 ± 0.5
LED(570)	23.3 ± 0.2	45.7 ± 0.4

*Values are reported as mean ± standard error of the mean.*

### Sampling and Measurements of Plant Morphological, Physiological and Optical Parameters

Chlorophyll a fluorescence was assessed from leaf number four using a portable HandyPEA continuous excitation chlorophyll fluorimeter (Hansatech, King’s Lynn, United Kingdom). First, light-adapted measurements to determine maximum operating efficiency of PSII photochemistry in the light [*F*_V_/*F*_M_′ = (*F*_M_′ – *F*_0_′)/*F*_M_′] were taken, then dark-adapted measurements were taken after 30 min of dark-adaptation using the manufacturer’s leaf clips and maximum quantum efficiency of PSII photochemistry in the dark [*F*_V_/*F*_M_ = (*F*_M_ – *F*_0_)/*F*_M_], non-photochemical quenching [NPQ = (*F*_M_ – *F*_M_′)/*F*_M_′] and performance index [PI = [1 – (*F*_0_/*F*_M_)/M_0_/V_J_] × [(*F*_M_ – *F*_0_)/*F*_0_] × [1 – V_J_)/V_J_)] were determined for four replicates.

The spectral properties of leaf number four were measured (Ocean Optics Jaz-SpectroClip-TR combined instrument, Ocean Optics, Dunedin, FL, United States) on the adaxial and abaxial leaf surface on day 30. Measurements were taken on the same leaf position in three plant replicates per treatment (on the right side of the midrib toward the leaf four apex). The leaf was illuminated by a standardized light source (Halogen lamp) through an optical fiber, and the transmitted and reflected light was analyzed with respect to its spectral composition.

**FIGURE 2:**
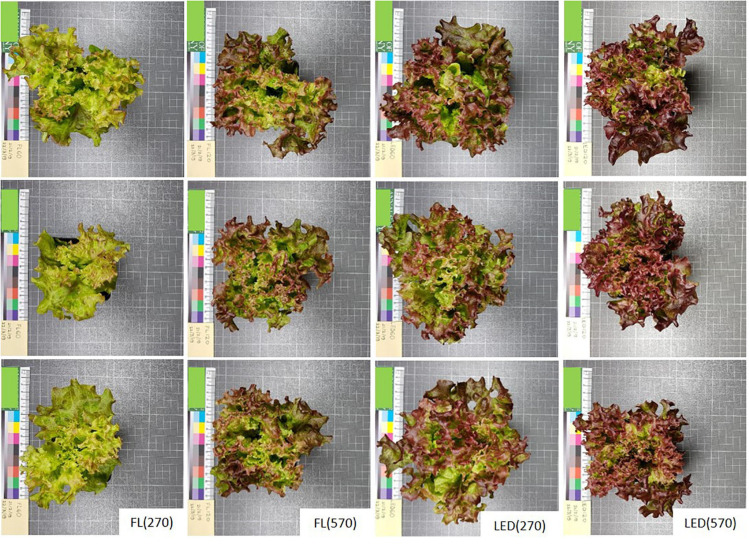
Representative pictures of three of the lettuce replicates treated with different light. Plants grown under fluorescent light (FL) and LEDs at two irradiance levels [270 and 570 μmol m^–2^ s^–1^) (FL(270), FL(570), LED(270), and LED(570)], showing differences in plant area, crinkliness and pigmentation. Pictures taken 30 days after sowing (DAS).

Rosette images (examples in Figure 2) were taken using a fixed focal length digital camera and fixed-lighting stand. Images were used for rosette area, measured as canopy cover, determination using the Shape descriptor plug in in ImageJ software (version 1.52a) (Schneider et al., 2012). Rosette shoots were harvested from just above the cotyledons node and immediately weighed to determine fresh weight (FW). Of the 12 plant replicates used to determine FW, eight were then placed in a paper bag and dried to constant weight at 60°C to determine DW.

A random selection of 3 plants were harvested for biochemical analyses at the end of the experiment (day 30). Fully expanded leaves, developmentally the third and fourth leaf, were excised, the midrib was removed and tissue immediately frozen in liquid nitrogen before storage at -80°C until analyzed. Prior to analysis, samples were freeze-dried and cold milled to a fine powder in an automated sample grinder (Labman Automation Ltd., Middlesbrough, United Kingdom) for 90 s at −70°C.

### Imaging of Leaf Disks by Light Microscopy and Transmission Electron Microscopy

Leaf disks of 1 cm^2^ were obtained from the fourth leaf (on the right side of the midrib toward the leaf apex) of four plant replicates and transferred to cold 2.5% glutaraldehyde in 0.1 M sodium cacodylate at pH 7.2, after vacuum infiltration discs were stored at 4°C. After a series of buffered washes, leaves were dehydrated in an aqueous alcohol (ethanol) series before being fixed in LR White (Hard grade) resin and were cut in 2–5 μm light microscopy (LM) sections on a Reichert-Jung Ultracut E Ultramicrotome, dried and blue stained. LM micrographs were taken using a Leica DM6000 microscope fitted with a Hitachi HV-D20 camera. Ultrathin 60 – 80 nm sections of interest were cut on a Ultramicrotome (Reichert-Jung Ultracut E) with a diamond knife (Diatome 21 Ultra 45°) and collected on Gilder GS2 × 0.5 3.05 mm diameter nickel or copper slot grids (Gilder Grids, Grantham, United Kingdom) float-coated with Butvar B98 polymer (Agar Scientific) films. transmission electron microscopy (TEM) sections were double-stained with uranyl acetate (Agar Scientific) and Reynold’s lead citrate (TAAB Laboratories Equipment Ltd., Aldermaston, United Kingdom) and observed using a JEOL JEM1010 transmission electron microscope (JEOL Ltd., Tokyo, Japan) at 80 kV. The resulting images were photographed using Carestream 4489 electron microscope film (Agar Scientific) and developed in Kodak D-19 developer. The derived negatives were scanned with an Epson Perfection V800 film scanner and converted to positive images (example images shown in Supplementary Figure 1). Leaf anatomy characteristics (leaf thickness, cell wall thickness and intercellular airspaces area) was measured on the images by digital analysis of the leaf-cross sections using ImageJ.

### Relative Water Content Determination

Leaf disks (1 cm^2^), cut from the right side of the midrib toward the leaf number four apex of four plant replicates, were used to determine the relative water content (RWC), calculated using the formula: [(FW-DW)/(TW-DW)]^∗^100, where FW is FW, DW is DW. TW, is turgid weight, which were obtained by leaving the leaf disk under distilled water in dark conditions for 24 h (Smart and Bingham, 1974).

### Estimation of Foliar Anthocyanin Content From Reflectance Spectra

Reflectance measurements, recorded on the leaf adaxial and abaxial surface on day 30, were used to assess red pigmentation due to the presence of anthocyanins. A three-band approach, mARI [(*R*_530__–__570_^–1^-*R*_690__–__710_^–1^)^∗^RNIR, where *R* was the reflectance at 530, 570, 690, and 790 nm and RNIR was the reflectance between 700 and 1000 nm] (Gitelson et al., 2006), was used to estimate leaf anthocyanin content. The red-edge band accounts for the variability derived from chlorophyll content and the NIR band for variability related to leaf structure and composition (Croft and Chen, 2018).

### Extraction and Quantification of Leaf Chlorophyll and Carotenoid Content

Lyophilized powdered leaf (15 mg) was extracted in three consecutive washes with 95% ethanol. After 48 h, absorbance of the collected extract was read at 470, 649, and 664 nm against the same amount of blank solution in a 96 well half area microplate ensuring a 1 cm pathlength using a UV/VIS spectrophotometer (UV 3100 PC Spectrophotometer, VWR, Belgium). Pigments concentration were determined using equations reported in Lichtenthaler and Buschmann (2001).

### Statistical Analysis

All the data were statistically analyzed using Microsoft Excel 2016 and R studio (R version 3.5.2 (2018-12-20), “Eggshell Igloo”) with packages: agricolae, car, ggplot2, Mendiburu (2010), Wickham (2016), and Fox and Weisberg (2019). For the effect of the light treatment on the measured parameters data were analyzed by one-way analysis of variance (ANOVA) and the means were compared by Least Significance Difference (LSD), at 5% significance level. The effects of two factors, “irradiance” and “light source,” and their interaction were tested by two-way analysis of variance (ANOVA) (Muggeo, 2003).

## Results

### Yield and Morphological Responses

Interaction effect was detected between irradiance and light source on the averaged FWs and DWs of red lettuce (*p* = 0.003 and 0.017, respectively). Light treatments had a significant effect on FW and DW (*p* = 0.000 and *p* = 7.0 × 10^–5^, respectively) of Lollo rosso lettuce growing in the same environment but under two different light sources at two different irradiances (Table 2). Increasing irradiance of FL significantly increased shoot FW (69%) and DW (98%) of “Lollo rosso.” Increasing irradiance under LED treatment did not significantly increase FW or DW and biomass values of both LED treatments were grouped with the higher FL treatment by *post hoc* test.

**TABLE 2:** Growth responses and leaf structural traits of Lollo rosso lettuce (30 DAS) growing under the same high and low irradiances of fluorescent, FL(270) and FL(570), and LED, LED(270), and LED(570), light.

**Treatments**	Rosette area** (cm^2^) (*n* = 3)	Fresh weight*** (g/head) (*n* = 12)	Dry weight*** (g/head) (*n* = 8)	Leaf thickness*** (mm) (*n* = 18)	Air spaces** (μm^2^) (*n* = 9)
FL(270)	181.9 ± 18.8^c^	10.2 ± 1.1^b^	0.62 ± 0.07^b^	0.18 ± 0.01^c^	1.89 ± 0.20^b^
FL(570)	202.8 ± 2.8^b,c^	17.3 ± 0.9^a^	1.23 ± 0.05^a^	0.22 ± 0.00^b^	4.11 ± 0.63^a^
LED(270)	259.3 ± 6.8^a^	18.6 ± 1.5^a^	1.25 ± 0.14^a^	0.21 ± 0.00^b^	2.67 ± 0.24^b^
LED(570)	229.5 ± 13.1^a,b^	17.6 ± 1.6^a^	1.27 ± 0.17^a^	0.24 ± 0.00^a^	2.33 ± 0.44^b^
Two-way	ANOVA				
Irradiance		*	*	***	*
Light source	**	**	**	***	
Interaction		**	*		

Values are reported as mean ± standard error of the mean. Different letters within columns indicate significant treatment differences at *P* < 0.05, as determined by Fisher’s least significant difference (LSD) test, with a > b > c. Significance codes (ANOVA): 0.000 “***,” 0.001 “**,” 0.01 “*.”

Rosette area (canopy cover) was significantly different between different light treatments (*p* = 0.004) (Table 2). Rosettes growing under treatment FL(270) were the smallest, resulting 12% smaller than rosettes grown under FL(570). In contrast, increasing irradiance under LEDs decreased rosette area. The largest rosettes of any treatments were from plants growing under LED(270), rosette area under the higher LED irradiance (LED570) was 13% lower but still greater than either FL treatment.

Light treatment had a significant effect on leaf thickness measured on cross-sections of the fourth leaf (*p* = 2.2 × 10^–5^). The thickest leaves were from plants growing under higher LED treatments [LED(570)] and the thinnest from FL(270) plants (Table 2 and Supplementary Figure 1). Light treatment did not have a significant effect on the leaf water status of Lollo rosso lettuce (Supplementary Table 1). Intercellular airspace doubled from FL(270) to FL(570) leaves which exhibited the highest values overall (*p* = 0.004) (Table 2).

### Chlorophyll Fluorescence, Leaf Optical Properties, and Pigments

Light treatment had a significant effect on the maximum operating efficiency of PSII photochemistry in the light, *F*_V_/*F*_M_′, and the maximum quantum efficiency of PSII photochemistry in the dark, *F*_V_/*F*_M_, (*p* = 0.017 and *p* = 0.030) (Table 3). The highest *F*_V_/*F*_M_′ was measured in leaves under FL(270), while the lowest was measured in leaves under LED(270). There was very little difference but LSD *post hoc* test separated measurements of *F*_V_/*F*_M_ in leaves grown in FL(270) from the rest of the treatments was slightly higher compared to an average of 0.84 from the other three treatments. No statistical difference was found for PI and NPQ (Supplementary Table 1). However, it was notable that the lowest levels of NPQ values were detected from leaves grown under treatment FL(270) and NPQ was two-fold higher in treatment LED(270).

**TABLE 3:** Chlorophyll fluorescence and chlorophyll and carotenoid pigment content of Lollo rosso lettuce growing under the same high and low irradiances of fluorescent, FL(270) and FL(570), and LED, LED(270), and LED(570), light for 30 days.

**Treatments**	*F*_V_/*F*_M_’* (*n* = 4)	*F*_V_/*F*_M_* (*n* = 4)	**Chlorophyll a (mg g^–1^) (*n* = 3)**	Chlorophyll b* (mg g^–1^) (*n* = 3)	Chlorophyll a:b** (*n* = 3)	Carotenoids* (mg g^–1^) (*n* = 3)
FL(270)	0.79 ± 0.01^a^	0.86 ± 0.00^a^	6.18 ± 0.07	2.04 ± 0.07^a,b^	3.03 ± 0.13^b^	1.46 ± 0.01^b^
FL(570)	0.75 ± 0.01^ab^	0.85 ± 0.00^ab^	5.99 ± 0.32	1.78 ± 0.09^c^	3.37 ± 0.02^a^	1.50 ± 0.07^b^
LED(270)	0.70 ± 0.04^b^	0.84 ± 0.01^b^	6.80 ± 0.15	2.30 ± 0.07^a^	2.96 ± 0.03^b^	1.55 ± 0.05^b^
LED(570)	0.72 ± 0.01^ab^	0.83 ± 0.01^b^	6.67 ± 0.23	1.99 ± 0.09^b,c^	3.35 ± 0.04^a^	1.72 ± 0.01^a^
Two-way	ANOVA					
Irradiance				**	**	*
Light source	*	*	*	*		**
Interaction						

Values are reported as mean ± standard error of the mean. Different letters within columns indicate significant treatment differences at *P* < 0.05, as determined by Fisher’s least significant difference (LSD) test, with a > b > c. Significance codes (ANOVA): 0.001 “**,” 0.01 “*.”

Light treatment did not have a significant effect on levels of the main reaction center pigment chlorophyll a (*p* = 0.079) but did have a significant effect on levels of the main pigment of the light harvesting complex, chlorophyll b, (*p* = 0.013) (Table 3). Two-way ANOVA reported a significant effect of both light source (*p* = 0.020) and irradiance (*p* = 0.008) on chlorophyll b content. The lowest chlorophyll b content was in FL(570) leaves while LED(270) leaves contained the greatest chlorophyll b content. The ratio of chlorophylls a and b of the LED grown plants was significantly affected by the light treatment (*p* = 0.007). Leaves grown under FL(270) and LED(270) treatments had the lowest ratio, i.e., greater light harvesting chlorophyll per reaction center, and leaves grown under the higher irradiance [FL(570) and LED(570)] had 11 and 13% greater chlorophyll a:b ratio. Light treatment had a significant effect on levels of the ancillary light harvesting and photoprotective carotenoid pigments (*p* = 0.014). Carotenoid content of leaves grown under the highest irradiance of LED treatment was significantly higher (15%) than the other three light treatments, which were statistically similar.

Two-way ANOVA demonstrated the light source had a significant effect on all the photosynthetic and pigment parameters measured except for chlorophyll a:b ratio and irradiance significantly affected pigments; chlorophyll b and carotenoid contents and chlorophyll a:b ratio. There was no detectable significant interaction effect on any of the measured parameters.

**FIGURE 3:**
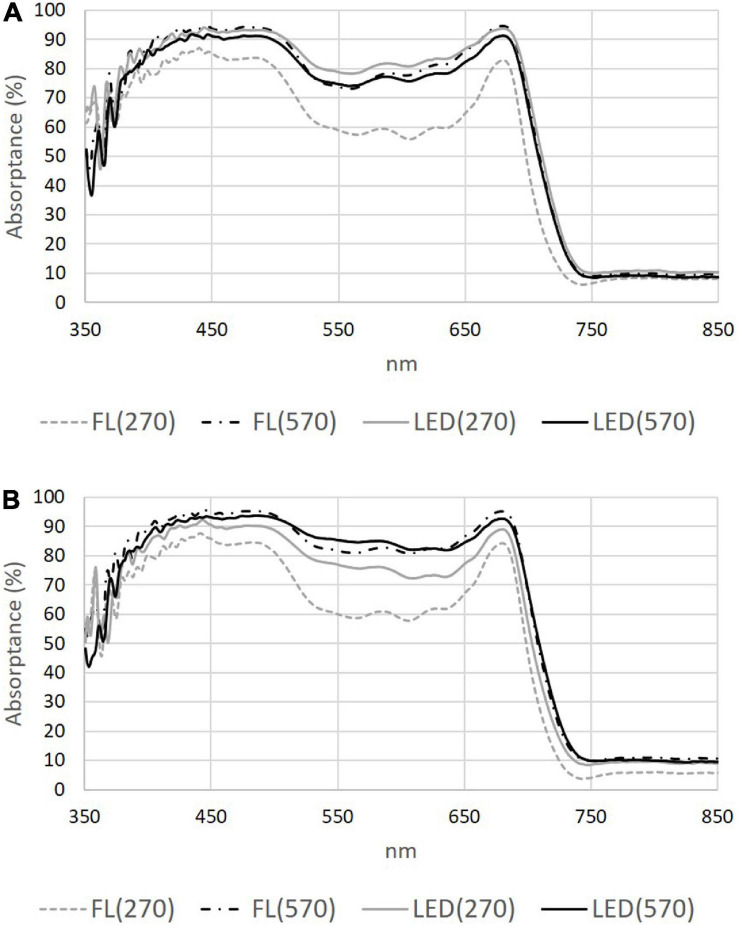
Leaf absorptance from 350 to 850 nm of red lettuce grown under different light sources (FL, compact fluorescence tubes and LED) at two irradiance levels (270 and 570 μmol m^–2^ s^–1^). **(A)** measurements taken on the adaxial side of leaf number four, **(B)** measurements taken on the abaxial side of leaf number four (*N* = 3). Absorptance was determined from reflectance and transmittance data measured on the leaf at 30 DAS using a Halogen lamp.

Absorptance measurements from the fourth leaf of “Lollo rosso” lettuce plants were influenced by light treatment (Figures 3A,B). Absorptance across the whole PAR region was affected by both light source (*p* = 0.001) and irradiance (*p* = 4.5 × 10^–5^). Leaf absorptance was significantly lower in FL(270) samples (∼10% less) than in the other three treatments in the PAR region (400–700 nm) (*p* = 3.3 × 10^–6^). Absorptance in the middle wavebands, exemplified by 560 nm, was significantly lower in FL(270) grown leaves (∼20% less) than all other leaves (*p* = 6.6 × 10^–6^). There was a significant interaction effect between light source and irradiance on the leaf absorptance at 560 nm (*p* = 0.001). Absorptance levels were similar from the adaxial and abaxial surfaces but the abaxial absorptance was always slightly lower. The modified anthocyanin reflectance index (mARI) of Lollo rosso lettuce leaves was significantly affected by both the light intensity (*p* = 0.026) and source (*p* = 0.040) (Table 4). In FL(270) leaves mARI values were half of those of all other treatments (*p* = 0.013), values increased in the order FL(570) < LED(270) < LED(570) but differences were not statistically significant. The normalized photochemical reflectance index [PRI_N_ = PRI/[RDVI^∗^(R_700_/R_670_)], where RDVI is the renormalized difference vegetation index and, R_670_ and R_700_ the reflectance at 670 and 700 nm, respectively (Zarco-Tejada et al., 2013) was statistically different between light source treatments (*p* = 1.1 × 10^–9^). PRI_N_ was almost five-fold higher in LED treated plants (Table 4).

**TABLE 4:** Percentage absorptance in the PAR region (400–700 nm) and at 560 nm and the modified anthocyanin reflectance index (mARI) and normalized photochemical reflectance index (PRI_N_) of cv. Lollo rosso lettuce leaves grown under the same high and low irradiances of fluorescent, FL(270) and FL(570), and LED, LED(270), and LED(570), light for 30 days.

**Treatments**	Absorptance PAR*** (%) (*n* = 6)	Absorptance at 560nm*** (%) (*n* = 6)	mARI* (*n* = 6)	PRI_N_*** (*N* = 6)
FL(270)	71.00 ± 1.94^b^	58.20 ± 2.52^b^	1.33 ± 0.13^b^	0.0025 ± 0.0000^b^
FL(570)	86.50 ± 1.54^a^	76.96 ± 2.36^a^	2.61 ± 0.27^a^	0.0035 ± 0.0002^b^
LED(270)	84.13 ± 1.58^a^	78.71 ± 2.00^a^	2.68 ± 0.53 ^a^	0.0153 ± 0.0021^a^
LED(570)	85.53 ± 1.41^a^	79.48 ± 2.63^a^	2.80 ± 0.20 ^a^	0.0125 ± 0.0015^a^
Two-way	ANOVA			
Irradiance	***	**	*	
Light source	**	***	*	***
Interaction	***	**		

Values are reported as mean ± standard error of the mean. Different letters within columns indicate significant treatment differences at *P* < 0.05, as determined by Fisher’s least significant difference (LSD) test, with a > b > c. Significance codes (ANOVA): 0.000 “***,” 0.001 “**,” 0.01 “*.”

## Discussion

Conventional incandescent sources for lighting are inefficient due to the significant production of heat rather than light, heat being an undesirable biproduct in most lighting situations particularly but not always in horticulture (Both et al., 2017). Improved efficiency was achieved from fluorescent lighting (FL) which in domestic systems was formulated as tubes and compact lighting (Mile, 2009) and fluorescent lamps have been used for over 50 years for plant growth (Thomas and Dunn, 1967; Tibbitts et al., 1983; Knight and Mitchell, 1988). Light emitting diode (LED) lighting is even more efficient in terms of reduced heat production and has additional benefits such as compact size, longer life span, greater luminous efficacy, affordable cost and allows greater control of spectra due to the narrow wavebands achievable from differently coated LEDs (Pattison et al., 2018). Thanks to the relatively rapid improvements and the possibility to adjust the spectral emission according to plant needs, LEDs are becoming an increasingly popular light source for plant growth both in greenhouse and closed CEs for industry and research (Mitchell et al., 2015). The light technology is being used for varied purposes including as growth light, to investigate light effects, to increase the daily light integral (DLI) or to environmentally modify the plant and is speeding up advances in horticulture (Bantis et al., 2018).

We made a direct comparison of two efficient light sources, fluorescent and LED, within the same CE cabinet. The light sources differed in their spectra and were applied at two light intensity levels (270 and 570 μmol m^–2^ s^–1^) to reduce the possibility that all treatments produced responses that were asymptotic in Lollo rosso lettuce.

The same lower irradiance (270 μmol m^–2^ s^–1^) provided by fluorescent and LED lights produced approximately twice the wet and DWs when delivered from LEDs. At equal PPF regimes, the major difference in the tested light treatments was in the proportions of different wavebands emitted by the two light sources (Figure 1). We detected no statistical difference in leaf temperature (23.2 ± 0.2°C) between treatments and thus the effect of the same photosynthetically photon flux densities (PPFDs) may be attributed to spectra perhaps through stimulation of photomorphogenic adaptations.

Optimized spectrum LEDs focused around the red and blue wavebands (red peaks at 634 and 665 nm and blue peak at 451 nm in our case) are highly efficient for plant growth (Matsuda et al., 2004; Zheng and Van Labeke, 2017), and also impact photomorphogenesis (Izzo et al., 2019). Rosette area (canopy cover) for example, a morphological adaptation supposedly resulting from the combination of multiple responses to light such as hypocotyl length, leaf angle and leaf shape (Hoenecke et al., 1992; Cammarisano et al., 2020), responded mainly to light source and antithetically under the tested light sources. If under FL rosettes tended to expand with increasing irradiance, under LEDs increasing irradiance produced more compacted rosettes. This response of rosettes impacts light interception, indicating that under low fluorescent light morphology alters to increase light interception, whereas under high LED interception is reduced probably due to light saturation of photosynthesis for Lollo rosso lettuce under the tested growth conditions.

Leaf anthocyanin content responded in parallel to rosette morphology and increased under high light. The photoprotective and antioxidant capacity of anthocyanins reduces light absorptance by chlorophyll and reduces photodamage by scavenging active oxygen resulting from the photo-excitation of chlorophyll (Gould et al., 2002; Kyparissis et al., 2007). Anthocyanin content, here estimated as mARI, increased under all light treatments compared with low FL. Bioactive compound accumulation increases in response to blue light (Ouzounis et al., 2015), while red and blue LEDs enhance both quality and yield in lettuce compared to FL lamps (Stutte et al., 2009). The FL source had major emission peaks around 560 and 610 nm and green–yellow radiation is reported to suppress lettuce growth (Dougher and Bugbee, 2001). Plants growing under low intensity FL had the lowest absorptance across the PAR region and when 560 nm was examined specifically (Table 4). Thus, the adaptive responses induced under low FL may be unable to efficiently utilize the available photons because this light is poorly intercepted. This is also in accordance with the plants grown under low FL appearing green indicating a lack of compounds absorbing in the mid-wavebands. “Weakly absorbed wavelengths” absorptance can be increased by lengthening of the light path in the leaf by the détour effect (Terashima et al., 2009). Plants under high FL produced the largest intercellular air spaces, a response that is characteristic of low light conditions (Ustin and Jacquemoud, 2020). The expansion of the intercellular air spaces increases light diffusion and the probability for a photon to be captured. The same was not observed in FL(270) leaves, supposedly to avoid negative consequences associated with air spaces such as reduced mesophyll conductance to CO_2_ (Gorton et al., 2003).

At higher irradiances there was no longer a difference between the biomass accumulated in plants growing under different light sources. Chlorophyll a:b ratio increased under higher intensity treatments regardless of the source [FL(570) and LED(570)] reflecting a decrease in light harvesting chlorophyll b in favor of reaction centers (Friedland et al., 2019). The observed adaptations in the chl a:b, responding mainly to light intensity this time, indicated an enhanced light use efficiency under higher PPFD. Additionally, leaf thickness was greater under LED(570), this response is known to increase in response to high light (Poorter et al., 2019) and is an adaptive strategy that enhanced water use efficiency (Yun and Taylor, 1986).

The increase in leaf carotenoid content in LED(570) confirmed the likely formation of excessive radiative energy and suggested these accessory pigments could enhance energy dissipation (Kress and Jahns, 2017). The higher carotenoid content was not reflected in higher NPQ suggesting that the photoprotective mechanisms induced including greater chlorophyll a:b ratio, carotenoid and anthocyanin content and reduced rosette area were sufficient to regulate light absorption and mitigate against phototoxicity derived from excess of light energy.

Our results show LEDs spectrum to potentially deliver more energy efficiently by producing twice the DW accumulated under the same photon flux emitted by FL. We hypothesize the light composition, and more precisely the differing proportions of blue, green–yellow, red and far-red photons, have differently excited the photosynthetic pigments of Lollo rosso lettuce altering its saturation threshold for yield and consequently determining the adaptive strategies implemented to enhance the use of the available light.

Thus, in efficiently exploiting LED light sources it is vital to identify the point at which the relationships between irradiance and desirable crop qualities become asymptotic in various environments in order to avoid energy wastage and especially negative influences on plant photosynthetic capacity and biomass accumulation. The inefficiency of the tested compact fluorescence tubes as a light source for Lollo rosso lettuce growth may also derive from the lack of a response that increases utilization of other available wavebands in this light. This presumably has little evolutionary impact but it may be that constitutively pigmented crops lack an appreciable benefit from artificial light sources.

We conclude that the stated increased efficacy of LED versus FL is a function of the irradiances compared and, at the higher irradiances compared here LEDs are no more efficient than fluorescent light. The presented results demonstrate the importance of the light source and its spectral quality plus the interaction with irradiance in controlling plant growth and quality, both in terms of morphology and nutritional content.

## Data Availability Statement

The original contributions presented in the study are included in the article/Supplementary Material, further inquiries can be directed to the corresponding author/s.

## Author Contributions

LC: conceptualization, methodology, investigation, formal analysis, writing – original draft, and writing – reviewing and editing. ID: funding acquisition, supervision. PR: funding acquisition, conceptualization, resources, writing – original draft, writing – reviewing, and editing, supervision. All authors contributed to the article and approved the submitted version.

## Conflict of Interest

The authors declare that the research was conducted in the absence of any commercial or financial relationships that could be construed as a potential conflict of interest.

## Supplementary Material

Supplementary Figure 1 Leaf cross-sections of red lettuce plants grown under two irradiance treatments (270 and 570 μmol m^−2^ s^−1^) emitted by two different light sources (Fluorescent light and LEDs). Images of leaf cross-sections are at the same magnification.

Supplementary Table 1 Effect of two irradiance treatments (270 and 570 μmol m^–2^ s^–1^) emitted by fluorescent tubes (FL) and LEDs on leaf disk relative water content (RWC), cell wall thickness, performance index (PI) and non-photochemical quenching (NPQ) of Lollo rosso lettuce at 30 DAS.

## References

[B1] Bantis F. Smirnakou S. Ouzounis T. Koukounaras A. (2018). Current status and recent achievements in the field of horticulture with the use of light-emitting diodes (LEDs). *Sci. Hortic.* 235 437–451. 10.1016/j.scienta.2018.02.058

[B2] Barber J. (2009). Photosynthetic energy conversion: natural and artificial. *Chem. Soc. Rev.* 38 185–196. 10.1039/b802262n 19088973

[B3] Becker C. Klaering H. P. Schreiner M. Kroh L. W. Krumbein A. (2014). Unlike quercetin glycosides, cyanidin glycoside in red leaf lettuce responds more sensitively to increasing low radiation intensity before than after head formation has started. *J. Agric. Food Chem.* 62 6911–6917. 10.1021/jf404782n 24382136 PMC4110108

[B4] Bensink J. (1971). *On Morphogenesis of Lettuce Leaves in Relation to Light and Temperature.* Wageningen: Veenman.

[B5] Bergstrand K. J. Mortensen L. M. Suthaparan A. Gislerød H. R. (2016). Acclimatisation of greenhouse crops to differing light quality. *Sci. Hortic.* 204 1–7. 10.1016/j.scienta.2016.03.035

[B6] Bjorkman O. Demmig-Adams B. (1995). “Regulation of photosynthetic light energy capture, conversion, and dissipation in leaves of higher plants,” in *Ecophysiology of Photosynthesis. Springer Study Edition*, Vol. 100 eds Schulze E. D. Caldwell M. M., (Berlin: Springer).

[B7] Both A. Bugbee B. Kubota C. Lopez R. G. Mitchell C. Runkle E. S. (2017). Proposed product label for electric lamps used in the plant sciences. *HortTechnology* 32 544–549. 10.21273/HORTTECH03648-16

[B8] Cammarisano L. Donnison I. S. Robson P. R. H. (2020). Producing enhanced yield and nutritional pigmentation in lollo rosso through manipulating the irradiance, duration, and periodicity of LEDs in the visible region of light. *Front. Plant Sci.* 11:82. 10.3389/fpls.2020.598082 33391308 PMC7775386

[B9] Carter G. A. Knapp A. K. (2001). Leaf optical properties in higher plants: linking spectral characteristics to stress and chlorophyll concentration. *Am. J. Bot.* 88 677–684. 10.2307/2657068 11302854

[B10] Carvalho S. D. Folta K. M. (2015). Expanding genetic potential with light environmentally modified organisms. *Crit. Rev. Plant Sci.* 2689 486–508. 10.1080/07352689.2014.929929

[B11] Croft H. Chen J. M. (2018). “Leaf pigment content,” in *Comprehensive Remote Sensing*, ed. Liang S., (Oxford: Elsevier). 10.1016/B978-0-12-409548-9.10547-0

[B12] Davis P. A. Caylor S. Whippo C. W. Hangarter R. P. (2011). Changes in leaf optical properties associated with light-dependent chloroplast movements. *Plant Cell Environ.* 34 2047–2059. 10.1111/j.1365-3040.2011.02402.x 21819411

[B13] Dougher T. A. O. Bugbee B. (2001). Evidence for yellow light suppression of lettuce growth. *Photochem. Photobiol.* 73:208. 10.1562/0031-86552001073<0208:efylso<2.0.co;2 11272736

[B14] Fox J. Weisberg S. (2019). *An R Companion to Applied Regression.* Available online at: https://socialsciences.mcmaster.ca/jfox/Books/Companion/.

[B15] Friedland N. Negi S. Vinogradova-Shah T. Wu G. Ma L. Flynn S. (2019). Fine-tuning the photosynthetic light harvesting apparatus for improved photosynthetic efficiency and biomass yield. *Sci. Rep.* 9 1–12. 10.1038/s41598-019-49545-8 31506512 PMC6736957

[B16] Gitelson A. A. Keydan G. P. Merzlyak M. N. (2006). Three-band model for noninvasive estimation of chlorophyll, carotenoids, and anthocyanin contents in higher plant leaves. *Geophys. Res. Lett.* 33 2–6. 10.1029/2006GL026457

[B17] Gorton H. L. Herbert S. K. Vogelmann T. C. (2003). Photoacoustic analysis indicates that chloroplast movement does not alter liquid-phase CO2 diffusion in leaves of *Alocasia brisbanensis*. *Plant Physiol.* 132 1529–1539. 10.1104/pp.102.019612 12857833 PMC167091

[B18] Gould K. S. McKelvie J. Markham K. R. (2002). Do anthocyanins function as antioxidants in leaves? Imaging of H2O2 in red and green leaves after mechanical injury. *Plant Cell Environ.* 25 1261–1269. 10.1046/j.1365-3040.2002.00905.x

[B19] Hoenecke M. E. Bula R. J. Tibbitts T. W. (1992). Importance of “blue” photon levels for lettuce seedlings grown under red-light-emitting diodes. *HortScience* 27 427–430. 10.21273/hortsci.27.5.427 11537611

[B20] Hoover W. H. (1937). *The Dependence of Carbon Dioxide Assimilation in a Higher Plant on Wave Length of Radiation*, Vol. 95. Washington, DC: Smithsonian Institution.

[B21] Izzo L. G. Arena C. De Micco V. Capozzi F. Aronne G. (2019). Light quality shapes morpho-functional traits and pigment content of green and red leaf cultivars of *Atriplex hortensis*. *Sci. Hortic.* 246 942–950. 10.1016/j.scienta.2018.11.076

[B22] Kalaji H. M. Schansker G. Brestic M. Bussotti F. Calatayud A. Ferroni L. (2017). Frequently asked questions about chlorophyll fluorescence, the sequel. *Photosynthesis Res.* 132 13–66. 10.1007/s11120-016-0318-y 27815801 PMC5357263

[B23] Knight S. L. Mitchell C. A. (1988). Effects of incandescent radiation on photosynthesis, growth rate and yield of waldmann’s green’ leaflettuce. *Sci. Hortic.* 35 37–49. 10.1016/0304-4238(88)90035-0 11539045

[B24] Kozai T. (2013). Resource use efficiency of closed plant production system with artificial light: concept, estimation and application to plant factory. *Proc. Japan Acad. B Phys. Biol. Sci.* 89 447–461. 10.2183/pjab.89.447 24334509 PMC3881955

[B25] Kress E. Jahns P. (2017). The dynamics of energy dissipation and xanthophyll conversion in arabidopsis indicate an indirect photoprotective role of zeaxanthin in slowly inducible and relaxing components of non-photochemical quenching of excitation energy. *Front. Plant Sci.* 8:2094. 10.3389/fpls.2017.02094 29276525 PMC5727089

[B26] Kusuma P. Pattison P. Bugbee B. (2020). From physics to fixtures to food: current and potential LED efficacy. *Hortic. Res.* 7:56. 10.1038/s41438-020-0283-7 32257242 PMC7105460

[B27] Kyparissis A. Grammatikopoulos G. Manetas Y. (2007). Leaf morphological and physiological adjustments to the spectrally selective shade imposed by anthocyanins in *Prunus cerasifera*. *Tree Physiol.* 27 849–857. 10.1093/treephys/27.6.849 17331903

[B28] Lichtenthaler H. K. Buschmann C. (2001). Chlorophylls and carotenoids: measurement and characterization by UV-VIS spectroscopy. *Curr. Protoc. Food Anal. Chem.* 1 F4.3.1–F4.3.8. 10.1002/0471142913.faf0403s01

[B29] Matsuda R. Ohashi-Kaneko K. Fujiwara K. Goto E. Kurata K. (2004). Photosynthetic characteristics of rice leaves grown under red light with or without supplemental blue light. *Plant Cell Physiol.* 45 1870–1874. 10.1093/pcp/pch203 15653806

[B30] McCree K. J. (1981). “Photosynthetically active radiation,” in *Physiological Plant Ecology I: Responses to the Physical Environment*, eds Lange O. L. Nobel P. S. Osmond C. B. Ziegler H., (Berlin: Springer). 10.1007/978-3-642-68090-8_3

[B31] Mendiburu F. (2010). Agricolae: statistical procedures for agricultural research. *R Package Version* 1 1–8.

[B32] Mile B. J. (2009). Lamps for improving the energy efficiency of domestic lighting. *Lighting Res. Technol.* 41 219–228. 10.1177/1477153509339610

[B33] Mitchell C. A. Dzakovich M. P. Gomez C. Lopez R. Burr J. F. Hernández R. (2015). Light-emitting diodes in horticulture. *Hortic. Rev.* 43 1–88. 10.1002/9781119107781.ch01

[B34] Muggeo V. M. R. (2003). Estimating regression models with unknown break-points. *Stat. Med.* 22 3055–3071. 10.1002/sim.1545 12973787

[B35] Niyogi K. K. (2000). Safety valves for photosynthesis. *Curr. Opin. Plant Biol.* 3 455–460. 10.1016/S1369-5266(00)00113-8 11074375

[B36] Ouzounis T. Parjikolaei B. R. Frette X. Rosenqvist E. Ottosen C. O. (2015). Predawn and high intensity application of supplemental blue light decreases the quantum yield of PSII and enhances the amount of phenolic acids, flavonoids, and pigments in *Lactuca sativa*. *Front. Plant Sci.* 6:19. 10.3389/fpls.2015.00019 25767473 PMC4341431

[B37] Pattison P. M. Tsao J. Y. Brainard G. C. Bugbee B. (2018). LEDs for photons, physiology and food. *Nature* 563 493–500. 10.1038/s41586-018-0706-x 30464269

[B38] Poorter H. Niinemets U. Ntagkas N. Siebenkas A. Maenpaa M. Matsubara S. (2019). A meta-analysis of plant responses to light intensity for 70 traits ranging from molecules to whole plant performance. *New Phytol.* 223 1073–1105. 10.1111/nph.15754 30802971

[B39] Reynolds S. G. (1970). Gravimetric meth of soil moisture determination. *J. Hydrol.* 11 258–273.

[B40] Ruban A. V. Berera R. Ilioaia C. Van Stokkum I. H. M. Kennis J. T. M. Pascal A. A. (2007). Identification of a mechanism of photoprotective energy dissipation in higher plants. *Nature* 450 575–578. 10.1038/nature06262 18033302

[B41] Schneider C. A. Rasband W. S. Eliceiri K. W. (2012). NIH image to imageJ: 25 years of image analysis. *Nature Methods* 9 671–675. 10.1007/978-1-84882-087-6_9 22930834 PMC5554542

[B42] Smart R. E. Bingham G. E. (1974). Rapid estimates of relative water content. *Plant Physiol.* 53 258–260. 10.1104/pp.53.2.258 16658686 PMC541374

[B43] Štroch M. Špunda V. Kurasová I. (2004). Non-radiative dissipation of absorbed excitation energy within photosynthetic apparatus of higher plants. *Photosynthetica* 42 323–337. 10.1023/B:PHOT.0000046149.97220.18

[B44] Stutte G. W. Edney S. Skerritt T. (2009). Photoregulation of bioprotectant content of red leaf lettuce with light-emitting diodes. *HortScience* 44 79–82. 10.21273/hortsci.44.1.79

[B45] Terashima I. Fujita T. Inoue T. Chow W. S. Oguchi R. (2009). Green light drives leaf photosynthesis more efficiently than red light in strong white light: revisiting the enigmatic question of why leaves are green. *Plant Cell Physiol.* 50 684–697. 10.1093/pcp/pcp034 19246458

[B46] Thapper A. Mamedov F. Mokvist F. Hammarstrom L. Styring S. (2009). Defining the far-red limit of photosystem II in Spinach. *Plant Cell Online* 21 2391–2401. 10.1105/tpc.108.064154 19700631 PMC2751953

[B47] Thomas A. S. Dunn S. (1967). Plant growth with new fluorescent lamps. *Planta* 212 208–212. 10.1007/BF00387484 24554213

[B48] Tibbitts T. W. Morgan D. C. Warrington I. J. (1983). Growth of lettuce, spinach, mustard, and wheat plants under for combinations of high-pressure sodium, metal halide, and tungsten halogen lamps at equal PPFD. *J. Am. Soc. Hortic. Sci.* 108 622–630.

[B49] Ustin S. L. Jacquemoud S. (2020). “How the optical properties of leaves modify the absorption and scattering of energy and enhance leaf functionality,” in *Remote Sensing of Plant Biodiversity*, eds Cavender-Bares J. Gamon J. A. Townsend P. A., (Cham: Springer). 10.1007/978-3-030-33157-3

[B50] Wickham H. (2016). *ggplot2, Elegant Graphics for Data Analysis.* New York, NY: Springer, 10.1007/978-3-319-24277-4

[B51] Yun J. I. Taylor S. E. (1986). Adaptive implications of leaf thickness for sun- and shade-grown abutilon theophrasti. *Ecol. Soc. Am.* 67 1314–1318. 10.2307/1938687

[B52] Zarco-Tejada P. J. González-dugo V. Williams L. E. Suárez L. Berni J. A. J. Goldhamer D. (2013). Remote sensing of environment a PRI-based water stress index combining structural and chlorophyll effects : assessment using diurnal narrow-band airborne imagery and the CWSI thermal index. *Remote Sensing Environ.* 138 38–50. 10.1016/j.rse.2013.07.024

[B53] Zheng L. Van Labeke M. C. (2017). Long-term effects of red- and blue-light emitting diodes on leaf anatomy and photosynthetic efficiency of three ornamental pot plants. *Front. Plant Sci.* 8:917. 10.3389/fpls.2017.00917 28611818 PMC5447751

[B54] Zhu X. Long P. S. Ort D. R. (2008). What is the maximum efficiency with which photosynthesis can convert solar energy into biomass? *Curr. Opin. Biotechnol.* 19 153–159. 10.1016/j.copbio.2008.02.004 18374559

