# Supplementary figures and images for: The Effect of Red & Blue Rich LEDs vs Fluorescent Light on Lollo Rosso Lettuce Morphology and Physiology
Source: Front Plant Sci. 2021 Feb 18;12:603411. doi: 10.3389/fpls.2021.603411 (PMC7930480; doi:10.3389/fpls.2021.603411)

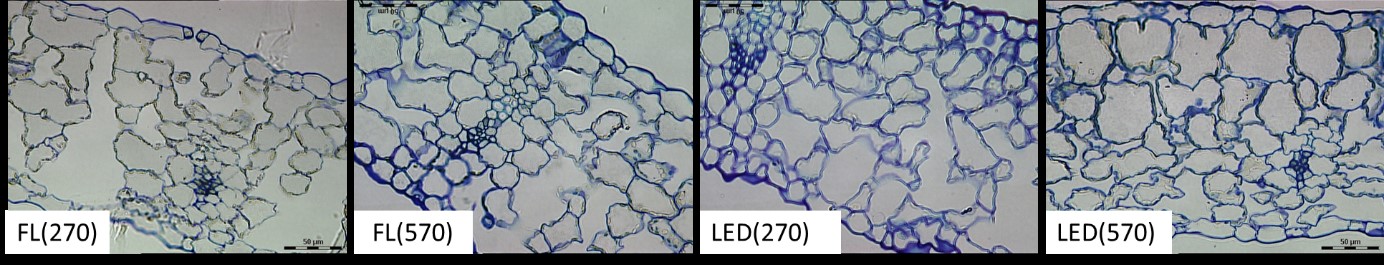

Supplement: Supplementary Figure 1 — Leaf cross-sections of red lettuce plants grown under two irradiance treatments (270 and 570 μmol m−2 s−1) emitted by two different light sources (Fluorescent light and LEDs). Images of leaf cross-sections are at the same magnification. [file Image_1.jpg]
