# Supplementary material for: The Effect of Red & Blue Rich LEDs vs Fluorescent Light on Lollo Rosso Lettuce Morphology and Physiology
Source: Front Plant Sci. 2021 Feb 18;12:603411. doi: 10.3389/fpls.2021.603411 (PMC7930480; doi:10.3389/fpls.2021.603411)
Supplement: Supplementary Table 1 — Effect of two irradiance treatments (270 and 570 μmol m–2 s–1) emitted by fluorescent tubes (FL) and LEDs on leaf disk relative water content (RWC), cell wall thickness, performance index (PI) and non-photochemical quenching (NPQ) of Lollo rosso lettuce at 30 DAS. [file Table_1.docx]

Table S1. Effect of two irradiance treatments (270 and 570 μmol m^-2^ s^-1^) emitted by fluorescent tubes (FL) and LEDs on leaf disk relative water content (RWC), cell wall thickness, performance index (PI) and non-photochemical quenching (NPQ) of Lollo rosso lettuce at 30 DAS.

| Treatments | Leaf disk DW  (g) (N=4) | Leaf disk RWC  (%) (N=4) | Cell wall  (μm) (N=9) | PI  (N=4) | NPQ  (N=4) |
| --- | --- | --- | --- | --- | --- |
| FL(270) | 0.95 ± 0.03 | 58.1 ± 7.6 | 0.62 ± 0.07 | 2.52 ± 0.52 | 0.25 ± 0.04 |
| FL(570) | 1.18 ± 0.09 | 44.3 ± 5.2 | 1.23 ± 0.05 | 3.14 ± 0.60 | 0.33 ± 0.05 |
| LED(270) | 1.23 ± 0.08 | 45.6 ± 3.9 | 1.25 ± 0.14 | 2.60 ± 0.45 | 0.62 ± 0.23 |
| LED(570) | 1.30 ± 0.13 | 49.4 ± 5.1 | 1.27 ± 0.17 | 3.72 ± 1.15 | 0.32 ± 0.08 |

Values are reported as mean ± standard error of the mean.
